# Resident Front Office Experience: A Systems-Based Practice Activity

**DOI:** 10.3885/meo.2008.T0000120

**Published:** 2008-05-28

**Authors:** Gary Sutkin, Christine K. Aronoff

**Affiliations:** * Department of Obstetrics, Gynecology, and Reproductive Sciences, University of Pittsburgh, Pittsburgh, PA, USA; † Department of Obstetrics and Gynecology, Texas Tech University Health Sciences Center, Lubbock, Texas, USA

**Keywords:** resident education, front office, systems-based practice

## Abstract

**Purpose:**

We set out to create and evaluate a systems-based practice experience designed to introduce residents to front office responsibilities and stimulate suggestions for front office improvements.

**Methods:**

On two occasions in 2002 and 2006, each resident in the Obstetrics and Gynecology Department was trained by a front office staff member for one day. The residents completed pre- and post-experience surveys, answered open-ended questions about their experience, and volunteered suggestions for improving the front office staff, and were evaluated by their precepting staff member.

**Results:**

All but two of 23 particpating residents participated enthusiastically. These residents perceived experiencing the staff as vital to the success of the practice, reported an increased sense of appreciation for the training of staff personnel, and were evaluated favorably.

**Conclusion:**

This program gave our residents an appreciation for the training and responsibilities of pivotal office staff and an opportunity to suggest improvements. This program also satisfied ACGME resident education requirements regarding systems-based practice.

Physicians interact daily with office staff, including but not limited to front desk receptionists, phone clerks, and chart room and laboratory employees. Resident physicians may have a cursory understanding of what these valuable employees do, yet few have ever worked side-by-side with them and therefore may have only a limited appreciation of their importance in daily activities. Many of these residents will someday be in the position of hiring, evaluating, and firing these employees and will rely on their abilities for a successful practice.

Our goal was to create a pilot project for our Department that would satisfy ACGME resident education requirements for systems-based practice.^1^ Our hypotheses were that such a pilot project would:

1. Introduce the resident physician to the training level and daily responsibilities of certain front office staff members, and
2. Stimulate the resident physician to generate specific improvements for the front office staff

Additionally, we hoped that this project would improve relationships between the residents and front office staff.

Prior to starting the project, we performed a Medline search and a Business Source Premier database search using combinations of the terms “physicians’ offices,” “internship” and “residency,” “graduate medical education,” and “undergraduate medical education.” We found two surveys of Family Medicine residency directors regarding the teaching of office practice to Family Medicine residents.^2^,^3^ Rose et al surveyed 421 Family Medicine residency directors about the practice management content within their curriculum.^2^ They found that managed care had a significant impact on their curriculum and advocated active learning strategies to better educate their residents about practice management. The majority of the teaching was done in a didactic format, although some residents worked in office settings or were made a part of quality assurance or utilization review programs. Kellerman conducted a similar survey of Family Medicine residency directors in 1983.^3^ That survey was notable for its identification of key topics within a practice management curriculum, including fees and billing, hiring personnel, the economic details of starting a practice, interacting with Medicare, Medicaid, and other third-party payers, organizing medical records, and designing patient flow templates.

We found one description of a similar program for medical students.^4^ Moser et al described a program at the University of Kansas-Wichita in which medical students worked with coding and billing personnel, lab technicians, or a phone nurse. The authors emphasized the importance of introducing the student to the staff in a positive way, so as to avoid any enmity. They asked their staff for feedback on the student, especially regarding interpersonal skills, and even required some students to be involved in a “limited quality assurance project.”

## Methods

The structure of our Front Office Experience Program was drafted by consensus of the Residency Program Director and the Staff Coordinator in the Department of Obstetrics and Gynecology at Texas Tech University Health Science Center (TTUHSC). The TTUHSC Department of Obstetrics and Gynecology Residency Training Program is a university-based four-year program that enrolls three residents per year. The resident clinic is generally staffed by 11 office staff, and on an average day the clinic cares for 100 patients, answers 300 phone calls, handles 200 charts, and reviews 40 lab test results.

The program was implemented on two separate occasions, during February and March, 2002 and again during March and April, 2006 with two completely different groups of residents in the Obstetrics and Gynecology Department of TTUHSC. All four years of residents participated. Each resident was assigned to follow either 1) the Patient Services Specialist responsible for checking patients in at the front desk, 2) the Patient Services Specialist responsible for assigning labs to physicians for review, 3) the Patient Services Specialist responsible for answering the phones or 4) the Chart Room Organizer for one day. Each resident spent one half-day with at least two of the above employees (two half-days or one full day total). The resident was relieved of his/her clinical duties for that day. The resident was told to take off his or her “doctor coat” and think of himself as a front office staff member. Specifically, when answering a patient's phone call asking a medical question, the resident was instructed to triage the phone call to an appropriate clinician. The resident was to dress professionally and not wear his or her white coat.

The office member taught the resident his or her job as if the office was training a new employee. The resident was expected to begin with direct observation and perform the office member's job under her direct supervision by the end of the day. The resident ate lunch with the office member in the break room instead of with the other residents in the physician dining room.

Pre-experience and post-experience surveys were given to the residents to evaluate their expectations and experience. The post-experience survey was identical to the pre-experience survey except for the addition of a summative question. All questions were scored using five-point Likert scales except for one ranking question and three open-ended questions about the resident's perceptions of the front office staff. The answers to these questions were reviewed and grouped into common themes by one of the authors (GS). The front office staff member was given a separate form to evaluate the resident's performance during the day. Percentages and mean scores were tabulated for descriptive purposes and paired T-tests were used to compare pre-experience with post-experience answers. IRB approval was obtained (submission reference #014546).

## Results

Twenty-three residents participated in the program, and each spent 8.5 hours total in the front office with one or two front office preceptors. Most residents brought their lunch since our office members usually do the same. Each resident completed both the pre- and post-surveys (N = 23, see Table 1 and 2). One resident offered prolonged and vociferous complaints about her participation but still participated reluctantly. On both the Pre- and Post- surveys, she gave a 1 for every score, except for the ranking question, which was numbered consecutively from 1 to 7, without any attention to order. Her Pre- and Post- survey answers were included in the final analysis (see Table 1); her rankings were excluded (see Table 2).

**Table 2:** Residents ranking of the relative importance of the following 7 characteristics pre- and post-experience*

	Mean Rank (order)	
Characteristic	Pre-experience	Post-experience
Good communication skills	2.4 (1)	2.6 (1)
Courteous/friendly	2.9 (2)	2.8 (2)
Organized	3.2 (3)	2.9 (3)
Appropriate scheduling of patients	4.3 (4)	4.0 (4)
Respect for the doctor	5.0 (5)	4.8 (5)
Forms relationships with patients	5.4 (6)	5.7 (7)
Facile with computers	5.6 (7)	5.3 (6)

*The residents ranked each characteristic from 1 to 7, where 1 = most important and 7 = least important N = 22; The Pre- and Post-answers of one vociferously and reluctantly participating resident who numbered the 7 characteristics consecutively (from 1 to 7, without any order) were excluded

Staff evaluation of each resident was completed for 21 of the 23 residents (N = 21, see Table 3). The two missing surveys included the above-mentioned resident and another resident, for whom an evaluation was not completed. A third resident who did not complain was given all ones (the lowest possible score) by his evaluator along with the statement “does not care.” His/her evaluation scores were also included in the final analysis.

**Table 3:** Staff Evaluations of Residents and Overall Experience*

Characteristic	Mean Score†
Promptness	4.3
Eager to learn/participate	4.5
Courtesy in person with patients	4.6
Courtesy over phone with patients	4.4
Asked questions when needed	4.3
Communicated clearly with other staff	4.5
Interacted effectively with other staff	4.7
Team Player	4.6
Good at the computer	4.3
Identified and solved problems	4.5
Improved as the half-day progressed	4.5
Did you enjoy this experience?	4.8

*All characteristics were scored on a Likert scale from 1 to 5, where 1 = poor, 2 = fair, 3 = average, 4 = good, and 5 = excellent; except the final question, where 1 = not at all, 2 = a little, 3 = average, 4 = more than average, 5 = very much

†Mean Scores include one resident who was given all 1's by his/ her precepting Front Office Staff member.

## Surveys of Residents: Pre- and Post- Survey questions

Surveys of the 23 residents reflect that, save for the vociferous resident, all believed the front office staff to be “important” or “vital” to the success of a practice. Nineteen out of 23 (83%) residents answered post-experience that the front office staff was “much more important” to the patients as their own medical care of patients. Two residents felt they were “much less important.” Front office staff's work ethic was rated as working “somewhat hard” or “very hard” by 17 residents (74%). Two residents (9%) scored their work ethic as “lazy.” Post-experience, seven residents (30%) thought the front office job was “very demanding,” and five residents (22%) thought it was “not very demanding.”

The residents’ mean score to only one question changed from pre-experience to post-experience: their perception of how well the front desk employees were trained significantly increased from 2.8 pre-experience to 3.5 post-experience (Table 1, question 4, p < 0.01).

Eight residents (35%) found the overall experience to be “very worthwhile.” Eleven residents (48%) found the experience to be “average worthwhile.” Four residents (17%) found the experience to be “not very worthwhile.” (question 9) Mean scores for all pre- and post-experience are listed in Table 1 contained in the Appendix.

## Surveys of Residents: Rankings

The residents’ interpretation of the five most important characteristics of front office staff did not change from the Pre-experience to the Post-experience survey. They were as follows: “good communication skills,” “courteous and friendly,” “organized,” “appropriate scheduling of patients,” and “respect for the doctor” which ranked fifth of seven choices. (see Table 2) None of their mean scores to these questions changed significantly from pre-experience to post-experience.

## Surveys of Residents: Open-ended questions

The three questions asking for written responses generated some interesting answers. When asked what improvements they would make in the front office, the most common suggestion involved the scheduling of appointments, volunteered by 9 residents. Four residents suggested cross-training the staff to perform the duties of other staff. Three suggested hiring more staff. Two suggested staggering lunch coverage. Other interesting singular suggestions included standardizing the front office dress code, moving the charts closer to the front desk, and sponsoring appreciation dinners.

When asked to identify the greatest limitations that the front office faces, five residents mentioned the time-constraints and multiple tasks required of the staff and the paucity of time they are able to spend with each patient. Five noted a lack of communication with the physicians. Two mentioned that the staff had to answer to the demands and requests of many individuals at once, and two suggested the hiring of more bilingual staff members.

When asked what they learned from the experience, 15 residents mentioned the jobs’ more technical aspects, such as copying insurance cards, identifying necessary patient information to enter into the computer, and collecting patient copay. Three mentioned surprise that delays in moving the patients to the rooms were not due to late arrivals or inefficient staff, but were instead due to inherent delays in the process, such as registering the patients or obtaining the charts. Three residents mentioned that our front office staff displayed good rapport with each other.

## Surveys of Front Office Staff Members:

The 21 staff evaluations (see Table 3) demonstrated favorable evaluations for all but the one resident who, as previously mentioned, was given all 1's. The other 20 residents received a score of “good” or “excellent” on 212 out of 220 possible scores (11 questions for each of the 20 residents), or 96% of possible scores. Mean scores for all 21 residents were between 4.3 and 4.8 for all 11 questions. Nineteen (90%) staff members rated that they “very much” enjoyed their experience. Although we did not attempt to measure resident-staff relationships, anecdotally, we noticed a general improvement following both administrations of the project.

## Discussion

Our pilot project successfully satisfied our two research hypotheses. The surveys reflect that the resident gained a deeper understanding of the training level and daily responsibilities of certain front office staff members, and our residents generated helpful improvements for the front office staff. The residents indicated that the experience was both positive and informative and that they consider the front office staff to be an important part of patient care and the functioning of the office.

This program is an example of an activity that can satisfy ACGME core competency requirements for a systems-based practice activity. The ACGME requires that “residents demonstrate an awareness of and responsiveness to the larger context and system of health care and the ability to effectively call on system resources to provide care that is of optimal value.”^5^

We were impressed with how enjoyable the experience was for the front office staff. They rated the experience almost invariably as very enjoyable. Our department has experienced significant staff turnover in the past, and we hope that such an exercise might enhance staff member job satisfaction and retention.

This exercise was stimulated in part by reports of residents blaming patient delays on an “inept” front office staff and staff members complaining that the residents did not respect them. The evaluations for almost all of the residents reflect that these relationships were positive after the experience. Anecdotally, we have heard many positive comments from the staff and a willingness to repeat the experience.

Social Psychology refers to the process in which one employee learns the viewpoint of another employee as role reversal.^6^ Role reversal can reduce competitiveness and perceived threat in the workplace by increasing understanding of a coworker's position and encouraging compromise.^7^ Game Theory teaches that trust and cooperation is enhanced when there is a perception of similarity between two individuals and that two players who cooperate instead of compete can both do better in a zero-sum game.^8^ A contentious office culture can often seem like a zero-sum game. We anticipate that by taking on the role of a Front Office Staff member, most residents develop a greater sense of trust for them.

Two of our residents participated unenthusiastically. We suspect it inevitable that some residents will meet this project with either vocalized or non-vocalized disdain. Such an exercise might be a useful way to identify residents who get along poorly with support staff so that their professionalism might be addressed before they graduate.

Our study was limited by not rotating the residents among each front office staff member. It is possible that the resident and staff survey responses were biased by their pairings. Additionally, the residents’ experience might have been improved had each been able to work in all areas of the front office, but time constraints prohibited this. Furthermore, our residency program is small relative to most Obstetrics and Gynecology residency programs. A larger program may have provided more robust survey results. This program could also be enhanced by the addition of didactic sessions on practice management, meant to compliment the in-office experience.

In summary, we offer a detailed description of an educational experience designed to introduce our residents to the roles and responsibilities of the front office staff and to satisfy ACGME requirements for a systems-based practice activity. The program was rated favorably by the vast majority of two full cohorts of residents. These results suggest that the overwhelming majority of residents will accept such a program as worthwhile and will benefit accordingly, and we hope to continue this program for future resident cohorts.

## Appendix

**Table 1:** Surveys of Residents: Likert-scale questions

Question	Possible answers (scale 1 to 5)	Pre-Experience answers	%	Post-Experience answers	%	p*	df
**1: How important is the front office staff to the success of your practice?**							
Not important at all (1)		1	4%	1	4%		
Of little importance (2)		0	0%	0	0%		
Somewhat important (3)		0	0%	0	0%		
Important to its success (4)		1	4%	1	4%		
Vital to its success (5)		21	91%	21	91%		
**Mean**		**4.8**		**4.8**		**1.00**	**22**
**2: How important is the front office staff in relation to patient care?**							
Much less important (1)		1	4%	2	9%		
Somewhat less important (2)		0	0%	0	0%		
Just as important (3)		2	9%	2	9%		
Somewhat more important (4)		0	0%	0	0%		
Much more important (5)		19	83%	19	83%		
No answer		1	4%	0	0%		
**Mean**		**4.6**		**4.5**		**0.33**	**22**
**3: I feel that the front office staff works:**							
Not hard at all – they are lazy		2	9%	2	9%		
(1)							
Less than average (2)		0	0%	1	4%		
Average (3)		3	13%	3	13%		
Somewhat hard (4)		9	39%	8	35%		
Very hard (5)		8	35%	9	39%		
No answer		1	4%	0	0%		
**Mean**		**4.0**		**3.9**		**0.58**	**22**
**4: Do you feel that the front office staff is adequately trained?**							
Not very well trained (1)		5	22%	3	13%		
less than average training (2)		0	0%	0	0%		
Average amount of training (3)		15	65%	11	48%		
More than average training (4)		0	0%	0	0%		
Very well trained (5)		3	13%	9	39%		
**Mean**		**2.8**		**3.5**		**<0.01**	**22**
**5: How demanding of a job is it for front office staff?**							
Not very demanding (1)		3	13%	5	22%		
Less than average demanding		0	0%	0	0%		
(2)							
Average demanding (3)		13	57%	11	48%		
More than average demanding		0	0%	0	0%		
(4)							
Very demanding (5)		7	30%	7	30%		
**Mean**		**3.3**		**3.2**		**0.33**	**22**
**9: Was this experience worthwhile?**							
Not very worthwhile (1)		4	17%				
Less than average worthwhile		0	0%				
(2)							
Average worthwhile (3)		11	48%				
More than average worthwhile		0	0%				
(4)							
Very worthwhile (5)		8	35%				
**Mean**		**3.3**		**NA**			
